# CPI-1189 protects neuronal cells from oxygen glucose deprivation/re-oxygenation-induced oxidative injury and cell death

**DOI:** 10.18632/aging.202528

**Published:** 2021-02-17

**Authors:** Yong-Jun Li, Yueli Zhan, Chengrui Li, Jianhong Sun, Chengliang Yang

**Affiliations:** 1 Department of Anesthesiology, Lianshui County People's Hospital, Lianshui, China; 2 Anxi Maternal and Child Health Hospital, Anxi, China; 3 Department of Anesthesiology, Affiliated Hospital of Yangzhou University, Yangzhou, China

**Keywords:** CPI-1189, neurons, oxygen glucose deprivation/re-oxygenation, oxidative injury, signaling

## Abstract

Oxygen glucose deprivation (OGD)/re-oxygenation (OGDR) induces profound oxidative injury and neuronal cell death. It mimics ischemia-reperfusion neuronal injury. CPI-1189 is a novel tumor necrosis factor alpha-inhibiting compound with potential neuroprotective function. Here in SH-SY5Y neuronal cells and primary murine cortical neurons, CPI-1189 pretreatment potently inhibited OGDR-induced viability reduction and cell death. In OGDR-stimulated neuronal cells, p38 phosphorylation was blocked by CPI-1189. In addition, CPI-1189 alleviated OGDR-induced reactive oxygen species production, lipid peroxidation, and glutathione consumption. OGDR-induced neuronal cell apoptosis was also inhibited by CPI-1189 pretreatment. Furthermore, in SH-SY5Y cells and cortical neurons, CPI-1189 alleviated OGDR-induced programmed necrosis by inhibiting mitochondrial p53-cyclophilin D-adenine nucleotide translocase 1 association, mitochondrial depolarization, and lactate dehydrogenase release to the medium. In summary, CPI-1189 potently inhibited OGDR-induced oxidative injury and neuronal cell death.

## INTRODUCTION

Ischemic stroke is a major cause of human morbidities and mortalities around the world [1, 2]. The prevalence of this disease is rising in recent years [1, 2]. It is therefore important to further understand the pathological mechanisms of neuronal cell injury in ischemic stroke [3, 4], and to develop novel therapy strategies [2, 5, 6].

In cultured neurons, oxygen and glucose deprivation (OGD) re-oxygenation (OGDR) procedure is applied to mimic ischemia-reperfusion injury [7–10]. Sustained OGD (over 1h) will disrupt mitochondrial functions, and when coupled with re-oxygenation, significant reactive oxygen species (ROS) would be produced to cause severe oxidative injury [9, 11]. These events would lead to protein damage, lipid peroxidation, DNA breaks, and eventually neuronal cell necrosis and apoptosis [9, 11].

CPI-1189 (4-acetamido-N-(tert-butyl)-benzamide) is a tumor necrosis factor alpha (TNFα)-inhibiting compound. It has displayed cytoprotective and anti-inflammatory actions in different cell culture and animal models [12–17]. In animal models of Parkinson's disease (PD) and AIDS dementia, CPI-1189 treatment attenuated the deterioration in cognitive and/or motor function with no relevant side effects [16, 17].

Studies have also implied that CPI-1189 represents a promising neuroprotective compound. As it can protect neuronal cells/primary neurons from various stimuli [12–17]. CPI-1189 was able to mitigate TNFα-induced cell apoptosis and quinolinic acid-induced cell necrosis [14, 16]. In addition, CPI-1189 alleviated cell death caused by supernatants from macrophages of patients with AIDS dementia [14, 16]. Furthermore, CPI-1189 inhibited p38 phosphorylation and suppressed interleukin 1β (IL1β)-induced neuronal cell death [15]. Whether CPI-1189 can protect neuronal cells from OGDR-induced oxidative injury remains unknown.

## RESULTS

### CPI-1189 protects neuronal cells from OGDR-induced cell death

**Figure 1:**
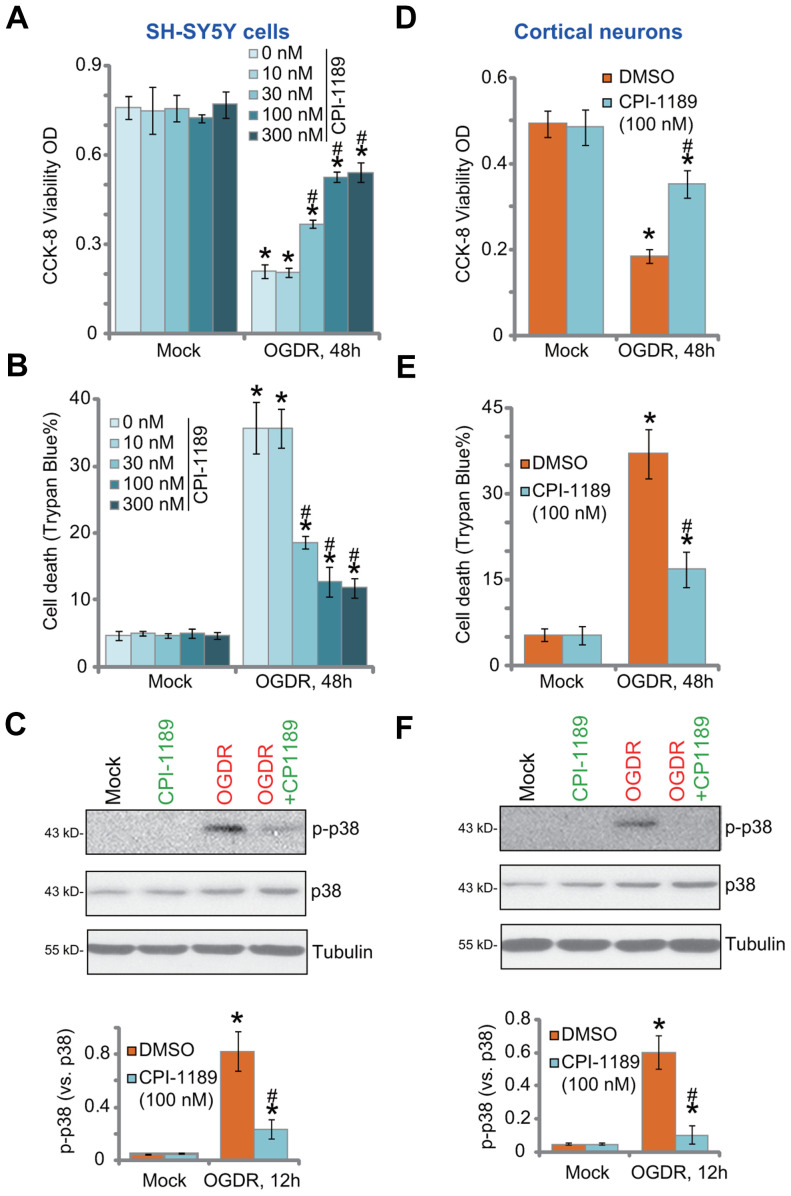
**CPI-1189 protects neuronal cells from OGDR-induced cell death.** SH-SY5Y neuronal cells (**A**–**C**) or primary murine cortical neurons (**D**–**F**) were pretreated for 1h with CPI-1189 (at applied concentrations) and subjected to OGDR procedure, cells were cultured for applied time periods, cell viability, cell death and p38 activation were tested by CCK-8 (**A**–**D**), Trypan blue staining (**B**–**E**) and Western blotting (**C**–**F**) assays, respectively. “Mock” stands for neuronal cells placed in norm-oxygenated regular medium containing glucose (same for all Figures). Quantified values were mean ± standard deviation (SD, n=5). * *P* < 0.05 *vs.* “Mock” cells. ^#^ *P* < 0.05 *vs.* cells with OGDR stimulation but “DMSO (0.1%)” pretreatment. Experiments were repeated three times, with similar results obtained.

SH-SY5Y neuronal cells were treated with CPI-1189 at gradually-increased concentrations from 10 to 300 nM. Cells were further cultured for 48h. Using CCK-8 cell viability and Trypan blue staining assays, we showed that CPI-1189, at tested concentrations, failed to significantly inhibit cell viability (Figure 1A) and induce cell death (Figure 1B). Next, OGDR was applied. SH-SY5Ycells were subjected to OGD for 4h, followed by re-oxygenation for another 48h. OGDR procedure led to over 70% viability (CCK-8 OD) reduction (Figure 1A) and significant cell death (increased Trypan blue ratio, Figure 1B). CPI-1189 pretreatment (for 1h) largely alleviated OGDR-induced cytotoxicity (Figure 1A, 1B). CPI-1189 displayed a concentration-dependent manner in protecting SH-SY5Y cells from OGDR (Figure 1A, 1B), especially at 30-300 nM (Figure 1A, 1B). It was however ineffective at 10 nM, the lowest concentration tested (Figure 1A, 1B). Since 100 nM of CPI-1189 displayed a significant effect against OGDR (Figure 1A, 1B), this concentration was selected for further studies.

OGDR-induced neuronal cell death is associated with p38 activation [18–20]. Inhibition of p38 can protect neuronal cells from OGDR-induced oxidative injury and cell death [18–20]. Studies have shown thatCPI-1189 was able to inhibit p38 activation to exert neuroprotective activity [12, 15]. Here we found that OGDR stimulation induced robust p38 activation (p38α Thr180/Tyr182 phosphorylation) in SH-SY5Y cells (Figure 1C). It was largely inhibited by CPI-1189 (100 nM) pretreatment (Figure 1C).

In primary murine cortical neurons, OGDR procedure induced potent viability (CCK-8 OD) reduction (Figure 1D) and cell death (Figure 1E). Both were attenuated by CPI-1189 (100 nM) pretreatment. OGDR-induced p38 activation was almost blocked by CPI-1189 (Figure 1F). CPI-1189 single treatment did not alter cell viability (Figure 1A–1D), cell death (Figure 1B–1E), or p38 activation (Figure 1C–1F) in SH-SY5Y cells and cortical neurons. These results showed that CPI-1189 protected neuronal cells from OGDR-induced cell death.

### CPI-1189 inhibits OGDR-induced oxidative injury in neuronal cells

**Figure 2:**
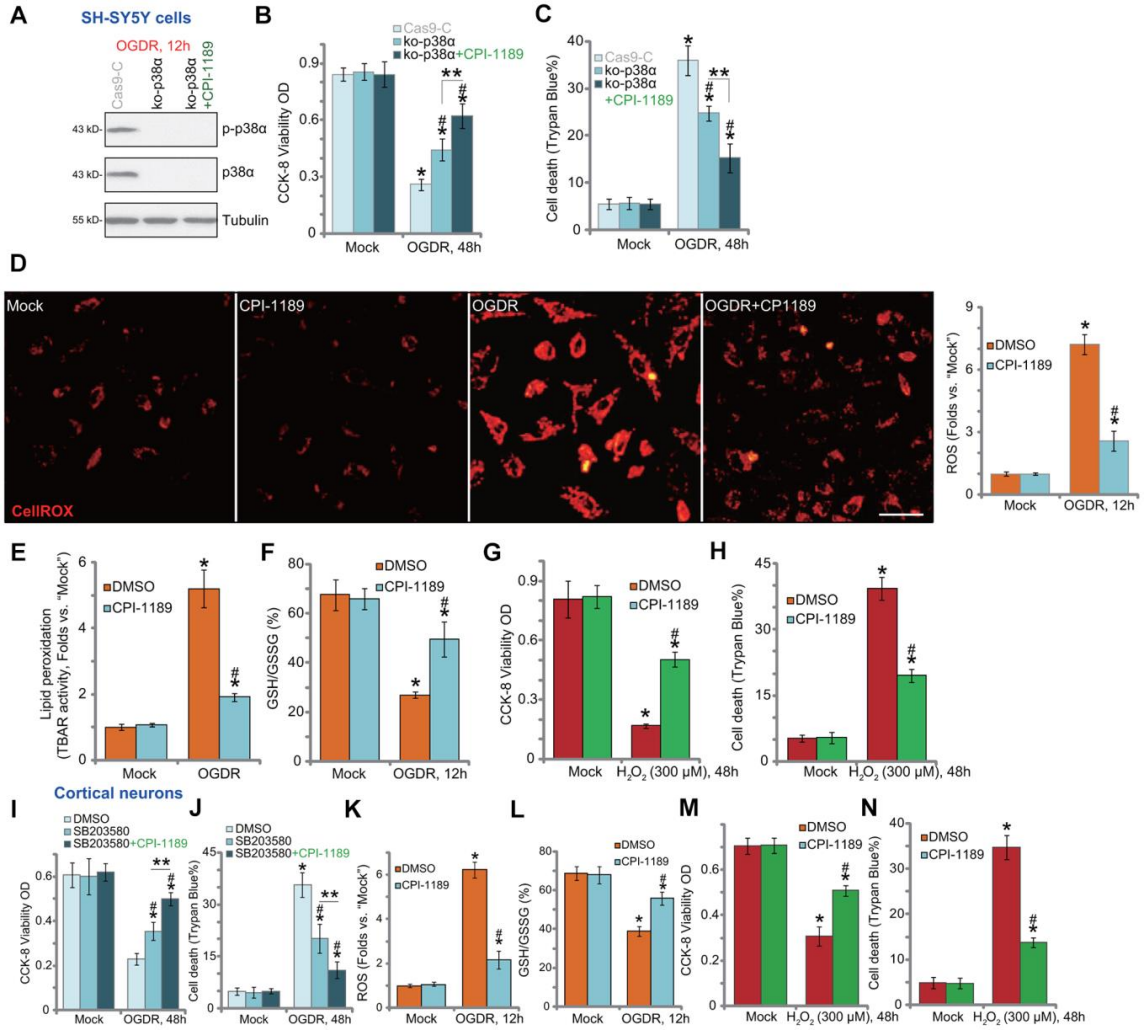
**CPI-1189 inhibits OGDR-induced oxidative injury in neuronal cells.** Stable SH-SY5Y cells with CRISPR/Cas9-p38α-KO-GFP (ko-p38α cells) were pretreated with or without CPI-1189 (100 nM, 1h pretreatment), control cells were transduced with the empty vector (“Cas9-C”), cells were subjected to OGDR procedure and cultured for applied time periods; Expression of listed proteins was shown (**A**); Cell viability and death were tested by CCK-8 (**B**) and Trypan blue staining (**C**) assays, respectively. SH-SY5Y cells (**D**–**H**) or primary murine cortical neurons (**K**–**N**) were pretreated for 1h with CPI-1189 (100 nM), followed by OGDR or hydrogen peroxide (H_2_O_2_, 300 μM) stimulation, cells were then cultured for applied time periods, cellular ROS contents (CellROX dye intensity, **D**, **K**), lipid peroxidation (by recording TBAR activity, **E**), and GSH/GSSG ratio (**F**–**L**) were tested. For cells with H_2_O_2_ stimulation, cell viability and death were tested by CCK-8 (**G**–**M**) and Trypan blue staining (**H**–**N**) assays, respectively. The primary murine cortical neurons were pretreated for 1h with SB203580 (5 μM) or plus CPI-1189 (100 nM), followed by OGDR stimulation and cells were then cultured for 48h; Cell viability and death were tested by CCK-8 (**I**) and Trypan blue staining (**J**) assays, respectively. * *P* < 0.05 *vs.* “Mock” cells. ^#^ *P* < 0.05 *vs.* cells with OGDR stimulation/H_2_O_2_ treatment but “DMSO (0.1%)” pretreatment. ** *P* < 0.05 (**B**, **C**, **I**, **J**). Quantified values were mean ± standard deviation (SD, n=5). Experiments were repeated three times, with similar results obtained. Scale bar= 100 μm (**D**).

To test whether p38 inhibition is the primary mechanism of CPI-1189-induced neuroprotection against OGDR, we utilized the CRISPR/Cas9 strategy to knockout p38α. As described, a CRISPR/Cas9-p38α-KO-GFP construct was transduced to SH-SY5Y cells. Single stable cells were established following GFP sorting and puromycin selection. These cells were namely as ko-p38α cells. As shown, p38α protein expression was depleted in ko-p38α cells (Figure 2A). OGDR-induced p38 activation, or p38α Thr180/Tyr182 phosphorylation, was blocked (Figure 2A). In ko-p38α SH-SY5Y cells, OGDR-induced viability reduction (Figure 2B) and cell death (Figure 2C) were alleviated. Significantly, CPI-1189 could still protect ko-p38α SH-SY5Y cells from OGDR (Figure 2B, 2C), indicating that p38-independent mechanisms should participate in CPI-1189-induced neuroprotection against OGDR.

OGDR is able to induce mitochondrial dysfunction and ROS production to mediate neuronal cell death [7–10, 20–22]. Conversely, antioxidants or other ROS scavenging strategies can protect neuronal cells from OGDR [7–10, 20–22]. By applying CellROX dye assay [23, 24], we found that OGDR stimulation in SH-SY5Y cells significantly increased cellular ROS contents (Figure 2D). It was largely inhibited by CPI-1189 (100 nM) pretreatment (Figure 2D). In addition, OGDR-induced lipid peroxidation (TBAR activity increase, Figure 2E) and GSH consumption (reflected by decreased GSH/GSSG ratio, Figure 2F) were inhibited by CPI-1189. These results implied that CPI-1189 inhibited OGDR-induced oxidative injury in SH-SY5Y cells. To mimic oxidative stress, hydrogen peroxide (H2O2) was added to cultured SH-SY5Y cells, resulting in significant viability reduction (Figure 2G) and cell death (Figure 2H). These were also inhibited by CPI-1189 pretreatment (Figure 2G, 2H).

In the primary murine cortical neurons, the p38 inhibitor SB203580 alleviated OGDR-induced cytotoxicity by restoring cell viability (Figure 2I) and inhibiting cell death (Figure 2J). Significantly, CPI-1189 offered additional neuroprotection against OGDR in cortical neurons (Figure 2I, 2J), indicating the existence of p38-independent mechanisms. Indeed, OGDR stimulation induced oxidative injury. It caused increase in CellROX intensity (Figure 2K) and GSH/GSSG ratio reduction (Figure 2L). OGDR-induced oxidative stress was potently inhibited by CPI-1189 (100 nM, 1h pretreatment) (Figure 2K, 2L). Furthermore, H2O2 induced significant viability (CCK-8 OD) reduction (Figure 2M) and cell death (Figure 2N) in cortical neurons, which were also attenuated by CPI-1189 pretreatment (Figure 2M, 2N). Collectively, CPI-1189 inhibited OGDR-induced oxidative injury in neuronal cells.

### CPI-1189 inhibits OGDR-induced apoptosis activation in neuronal cells

**Figure 3:**
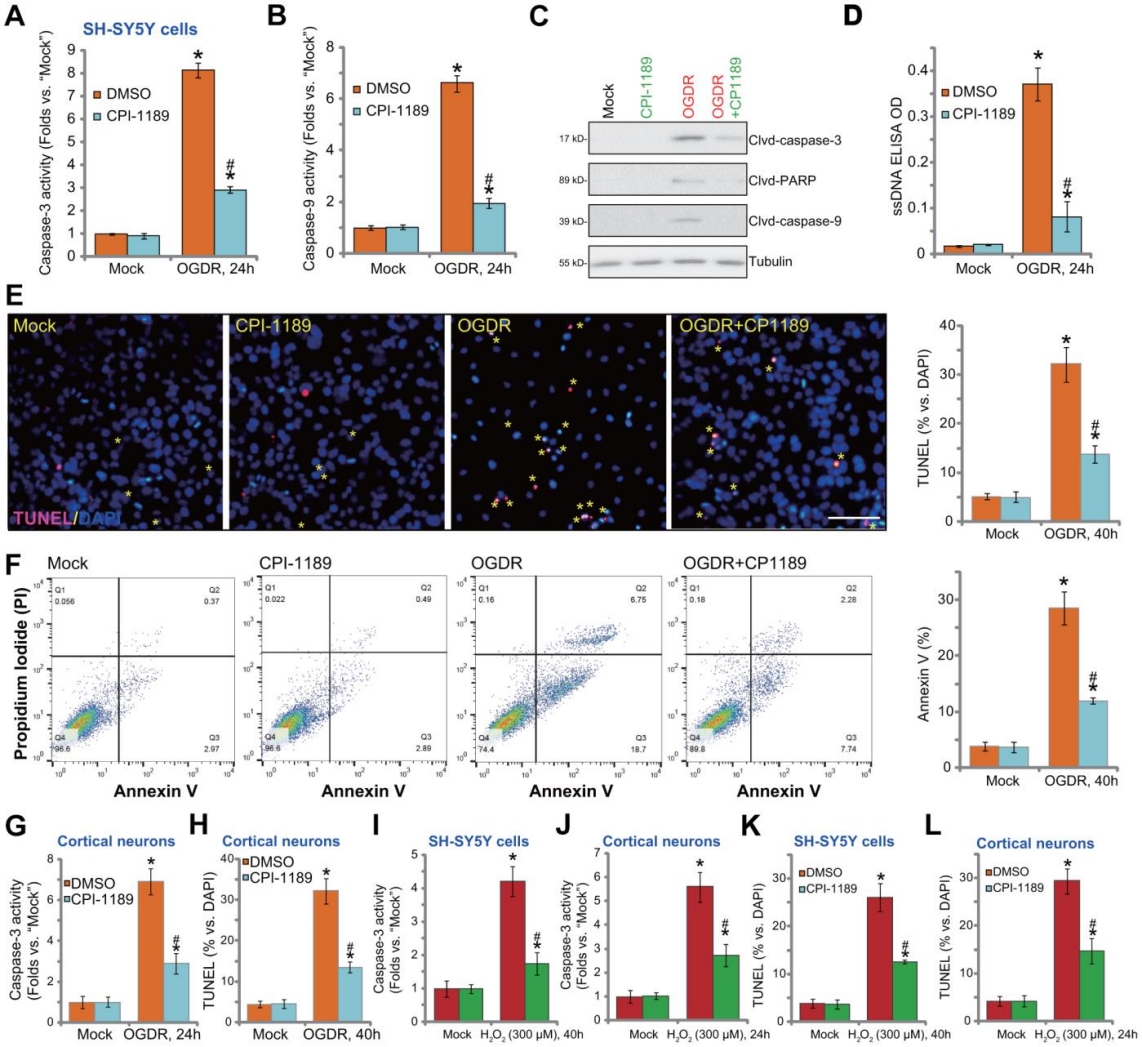
**CPI-1189 inhibits OGDR-induced apoptosis activation in neuronal cells.** SH-SY5Y neuronal cells (**A**–**F**, **I**–**K**) or primary murine cortical neurons (**G**, **H**, **J**–**L**) were pretreated for 1h with CPI-1189 (100 nM) and stimulated with OGDR or hydrogen peroxide (H_2_O_2_, 300 μM), cells were cultured for applied time periods, caspase activation and cell apoptosis were tested by the assays mentioned in the text. Quantified values were mean ± standard deviation (SD, n=5). * *P* < 0.05 *vs.* “Mock” cells. ^#^ *P* < 0.05 *vs.* cells with OGDR stimulation/H_2_O_2_ treatment but “DMSO (0.1%)” pretreatment. Experiments were repeated three times, with similar results obtained. Scale bar= 100 μm (**E**).

OGDR-induced oxidative injury would lead to neuronal cell apoptosis [21, 22, 25, 26]. We thus tested the potential effect of CPI-1189 on cell apoptosis. Following OGDR stimulation, caspase-3 activity (Figure 3A) and caspase-9 activity (Figure 3B) were significantly increased in SH-SY5Y cells. In addition, cleavages of caspase-3, PARP (caspase-3’s substrate), and caspase-9 were detected in OGDR-stimulated cells (Figure 3C). Single strand DNA (ssDNA) accumulation was investigated (indicating DNA breaks, Figure 3D). These results implied activation of mitochondrial apoptosis cascade in OGDR-stimulated SH-SY5Y cells. Importantly, CPI-1189 (100 nM, 1h pretreatment) potently inhibited OGDR-induced caspase-3/-9 activation (Figure 3A–3C) and ssDNA accumulation (Figure 3D) in SH-SY5Y cells.

Further confirming apoptosis activation in SH-SY5Y cells, TUNEL-positive nuclei (labeled with yellow stars) ratio was significantly increased following OGDR stimulation (Figure 3E). Also, cells with positive Annexin V staining were increased after OGDR (Figure 3F). CPI-1189 pretreatment largely attenuated OGDR-induced apoptosis activation in SH-SY5Y cells (Figure 3E, 3F). CPI-1189 single treatment failed to induce caspase (Figure 3A–3D) and apoptosis (Figure 3E, 3F) activation in SH-SY5Y cells.

In primary murine cortical neurons, OGDR stimulation increased caspase-3 activity (Figure 3G) and TUNEL- positive nuclei ratio (Figure 3H), which were largely attenuated byCPI-1189 (100 nM, 1h pretreatment, Figure 3G, 3H). As the positive control, H2O2 was utilized to increase caspase-3 activation (Figure 3I, 3J) and TUNEL-positive nuclei ratio (Figure 3K, 3L) in SH-SY5Y neuronal cells and primary neurons. Importantly, CPI-1189 pretreatment largely inhibited H2O2-induced apoptosis activation in neuronal cells (Figure 3I–3L). Thus, CPI-1189 inhibited OGDR-induced apoptosis activation in neuronal cells.

### CPI-1189 inhibits OGDR-induced programmed necrosis in neuronal cells

**Figure 4:**
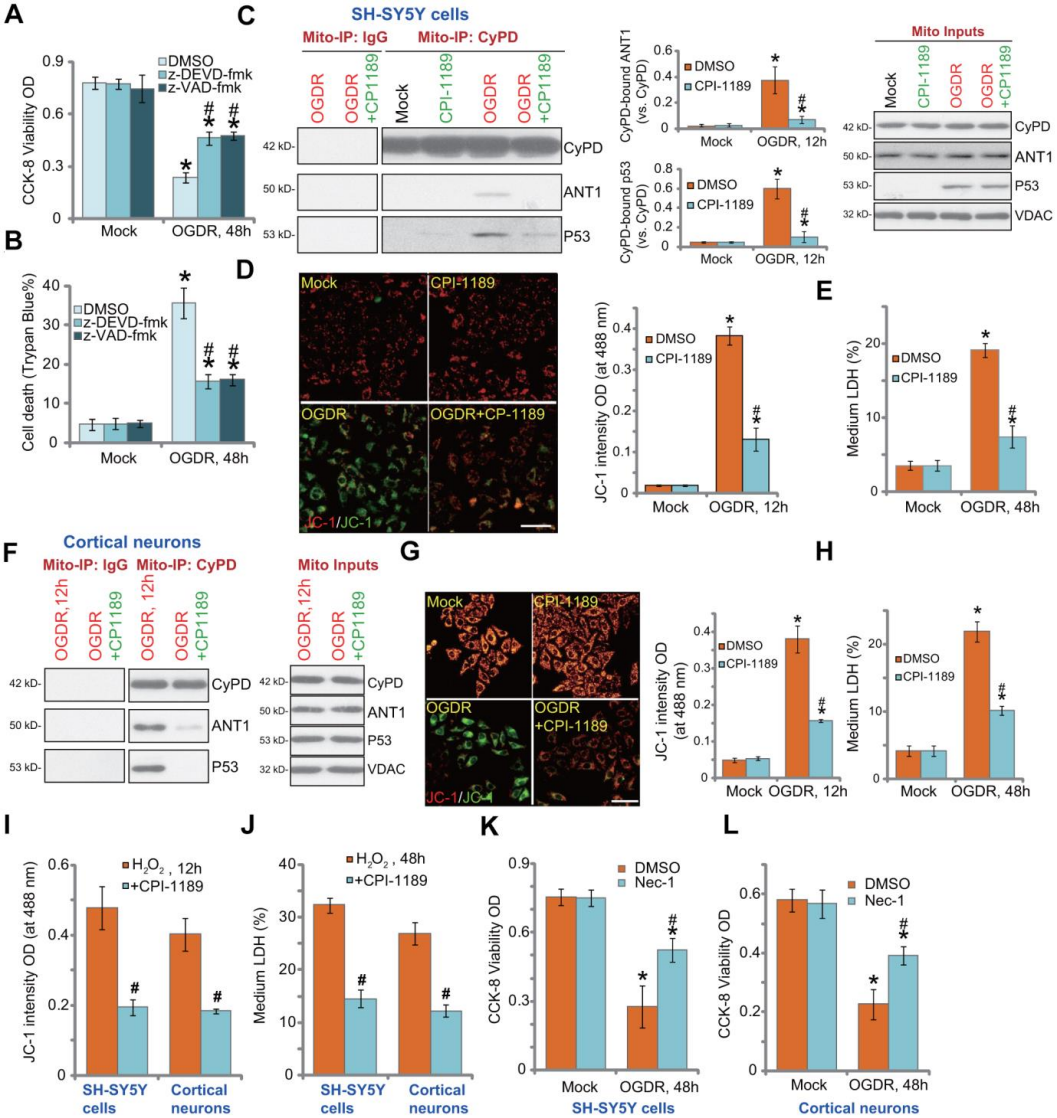
**CPI-1189 inhibits OGDR-induced programmed necrosis in neuronal cells.** SH-SY5Y cells were pretreated for 1h with z-DEVD-fmk or z-VAD-fmk (each at 50 μM), followed by OGDR stimulation; Cells were cultured for another 48h, cell viability and death were tested by CCK-8 (**A**) and Trypan blue staining (**B**) assays, respectively. SH-SY5Y neuronal cells (**C**–**E**) or primary murine cortical neurons (**F**–**H**) were pretreated for 1h with CPI-1189 (100 nM) and treated with OGDR, cells were cultured for applied time periods, mitochondrial p53-CyPD-ANT1 association (“Mito-IP: CyPD”) and their expression (“Mito Inputs”) were tested (**C**–**F**); Mitochondrial depolarization and cell necrosis were tested by JC-1 dye assay (**D**–**G**) and medium LDH release (**E**–**H**), respectively. SH-SY5Y neuronal cells or primary murine cortical neurons were pretreated for 1h with CPI-1189 (100 nM) and stimulated with hydrogen peroxide (H_2_O_2_, 300 μM); Cells were cultured for applied time periods, mitochondrial depolarization (**I**) and cell necrosis (**J**) were tested similarly. SH-SY5Y cells or primary cortical neurons were pre-treated for 1 hour with 25 μM of necrostatin-1 (“Nec-1”), followed by OGDR stimulation and cells were then cultured for 48h; Cell viability was tested by CCK-8 assays (**K**, **L**). Quantified values were mean ± standard deviation (SD, n=5). * *P* < 0.05 *vs.* “Mock” cells. ^#^ *P* < 0.05 *vs.* cells with OGDR stimulation/H_2_O_2_ treatment but “DMSO (0.1%)” pretreatment. Experiments were repeated three times, with similar results obtained. Scale bar= 100 μm (**D**–**G**).

In SH-SY5Y cells, the caspase-3 inhibitor z-DEVD-fmk and the pan caspase inhibitor z-VAD-fmk were only alleviated and not abolished OGDR-induced viability reduction (Figure 4A) and cell death (Figure 4B). Besides apoptosis studies have shown that OGDR can simultaneously induce programmed necrosis in neuronal cells [22, 25]. In OGDR-treated SH-SY5Y cells, p53 translocated to mitochondria (Figure 4C, “Mito Inputs”) and immunoprecipitated with CyPD and ANT1 (Figure 4C, “Mito-IP”), two key components of mPTP [27, 28]. Furthermore, mitochondrial depolarization, evidenced by mitochondrial JC-1 green monomers accumulation (Figure 4D), was detected in OGDR-treated SH-SY5Y cells. It was followed by medium LDH release (Figure 4E). These results confirmed the activation of mitochondrial programmed necrosis cascade in OGDR-treated SH-SY5Y cells (see other studies reporting the same cascade [28, 29]). Significantly, CPI-1189 pretreatment largely attenuated OGDR-induced programmed necrosis activation in SH-SY5Y cells, inhibiting p53-CyPD-ANT1 association (Figure 4C), mitochondrial depolarization (Figure 4D), and LDH release to the medium (Figure 4E).

Similar results were obtained in primary murine cortical neurons. CPI-1189 pretreatment inhibited OGDR-induced mitochondrial p53-CyPD-ANT1 association (Figure 4F), JC-1 green monomers accumulation (Figure 4G), and cell necrosis (Figure 4H). CPI-1189 by itself, as expected, did not induce programmed necrosis cascade in SH-SY5Y cells and cortical neurons (Figure 4C-H). Following H2O2 stimulation, mitochondrial depolarization (JC-1 green monomers accumulation) was detected in SH-SY5Y cells and primary cortical neurons (Figure 4I). Cell necrosis, evidenced by medium LDH release, was detected as well (Figure 4J). CPI-1189 pretreatment inhibited H2O2-induced actions above in SH-SY5Y cells and cortical neurons (Figure 4I, 4J). Therefore CPI-1189 inhibited OGDR-induced programmed necrosis in neuronal cells. Necrostatin-1 (Nec-1) is a specific necrosis inhibitor and it directly blocks receptor-interacting serine/threonine-protein kinase 1/3 (RIPK1/3) [30]. Further supporting our hypothesis, we found that Nec-1 reduced OGDR-induced viability reduction in SH-SY5Y cells (Figure 4K) and primary cortical neurons (Figure 4L).

## DISCUSSION

CPI-1189 is a compound clinically evaluated as a potential therapy for AIDS patients with dementia [13, 17]. Recent *in vitro* and *in vivo* studies have proposed the potential neuroprotective property of this compound [14–16]. Supporting its potential function in protecting neurons, we found that CPI-1189 pretreatment, at only nM concentrations, potently inhibited OGDR-induced viability reduction and death in SH-SY5Y cells and murine cortical neurons. CPI-1189 blocked OGDR-induced p38 activation. This compound was also neuroprotective in p38α-KO SH-SY5Y cells and p38-inhibited cortical neurons, indicating the possibility of p38-independent mechanisms.

OGDR-induced neuronal cell injury is associated with oxidative stress [9, 11, 21, 22]. OGD can severely impair mitochondrial functions, and ROS produced by re-oxygenation would then cause significant oxidative stress, DNA breaks, protein damage, inflammation, lipid peroxidation, and eventually neuronal cell death. Conversely, ROS scavenging is able to protect neuronal cells from OGDR [21, 22, 25].

Di et al., showed that in neuronal cells, microRNA-613 silencing upregulated its target SphK2 and inhibited OGDR-induced oxidative stress [21]. The same group reported that the SphK1 activator K6PC-5 provoked SphK1-Nrf2 signaling to inhibit OGDR-induced oxidative injury in neuronal cells [22]. Zhang et al., showed that plumbagin improved OGDR-induced SH-SY5Y cell injury by inhibiting ROS [31].

In the present study, we show that CPI-1189-induced anti-OGDR activity was associated with ROS scavenging. CPI-1189 largely inhibited OGDR-induced ROS production, lipid peroxidation, and GSH consumption in SH-SY5Y cells and cortical neurons. Importantly, OGDR-induced neuronal cell apoptosis, the consequence of oxidative injury [21, 22, 25], was inhibited by CPI-1189 as well. Therefore, ROS scavenging should be one important mechanism of CPI-1189 protecting against OGDR.

Besides apoptosis OGDR can also provoke programmed necrosis in neuronal cells. Wang et al., found that OGDR induced NKILA (NF-κB Interacting LncRNA) upregulation to promote neuronal cell necrosis [25]. SphK1 activation by K6PC-5 inhibited OGDR-induced programmed necrosis in neuronal cells [22]. Here in SH-SY5Y cells and murine cortical neurons, CPI-1189 suppressed OGDR-induced programmed necrosis by inhibiting mitochondrial p53-CyPD-ANT1 association, mitochondrial depolarization, and LDH release to the medium. These results suggested a novel mechanism (inhibition of programmed necrosis) of anti-OGDR by CPI-1189. Future studies with concurrent inhibition of OGDR-induced cell necrosis and apoptosis should explain the superior neuroprotective activity by CPI-1189.

## MATERIALS AND METHODS

### Chemicals and reagents

CPI-1189 was provided by Selleck (Shanghai, China). Antibodies were obtained from Santa Cruz Biotechnology (Santa Cruz, CA, USA). DMSO, caspase-3 inhibitor z-DEVD-fmk, hydrogen peroxide (H_2_O_2_), necrostatin-1 (“Nec-1”), SB203580, the pan caspase inhibitor z-VAD-fmk, and puromycin were provided by Sigma-Aldrich (St. Louis, MO, USA).

### Cell culture

SH-SY5Y neuronal cells were provided by Dr. Di [32] and were cultured as described [32]. SH-SY5Y cells were differentiated by the incubation in BDNF plus glutamine medium (serum free) [32]. The primary murine cortical neurons were also provided by Dr. Di, and were cultured using previously described protocols [32]. At day-10 (DIV-10), over 95% of cells were cortical neurons. The protocols of using primary murine cells were approved by the Ethics Committee and IACUC of authors’ institution.

### Cell viability

Cell Counting Kit-8 (CCK-8, Dojindo Laboratories, Kumamoto, Japan) was utilized to test cell viability. Neuronal cells were seeded into 96-well plates at 4, 000 cells per well. After treatment, neuronal cells were incubated with CCK-8 reagent for 3h. In each well, CCK-8 optical density (OD) was tested at 450 nm.

### Cell death

Neuronal cells were seeded into 96-well plates at 4, 000 cells per well. Following treatment, dead cells were positively stained with Trypan blue. The ratio was recorded by an automatic cell counter (Roche, Shanghai, China).

### Lactate dehydrogenase (LDH) assay

Neuronal cells were seeded into six-well plates at 1×10^5^ cells per well. With the applied treatment, LDH contents in culture medium were analyzed through a two-step enzymatic reaction LDH assay kit (Takara, Tokyo, Japan), which were then normalized to total LDH contents.

### OGD/re-oxygenation

As reported [7], neuronal cells were placed in an airtight chamber with a continuous flux of gas (95% N_2_/5% CO_2_). The chamber was sealed and the cells were incubated under OGD for 4h. Cells were then re-oxygenated (OGDR) and cultured in regular medium for applied time period. “Mock” neuronal cells were placed in norm-oxygenated with regular medium containing glucose.

### Lipid peroxidation assay

Following treatment, thiobarbituric acid reactive substances (TBAR) assay was carried out to examine the cellular lipid peroxidation contents. The detailed protocol was reported before [21, 33].

### Western blotting

Protocols for Western blotting were described previously [34]. In brief, 30 μg protein lysates per treatment were loaded to 10-12% SDS-PAGE gels and transfected to PVDF blots. The blots were then blocked and incubated with the applied primary and secondary antibodies. ECL reagents were utilized to examine the targeted protein band. The ImageJ software (NIH) was utilized to quantify the protein band.

### Caspase-3/-9 activity

Previously describe protocol was used [7, 32]. In brief 20 μg of cytosolic protein lysates from neuronal cells with applied treatment were incubated with caspase-3/-9 substrate in the assay buffer [7]. Substrates were conjugated with 7-amido-4-(trifluoromethyl)-coumarin (AFC) (Calbiochem-EMD Millipore). An Fluoroskan Ascent FL machine was utilized to quantify the intensity of released AFC under 355 nm excitation and 525 nm emission.

### TUNEL (terminal deoxynucleotidyl transferase dUTP nick end labeling) assay

Neuronal cells were seeded into12-well plates (at 5 × 10^4^ cells per well). Following treatment, a TUNEL *In Situ* Cell Death Detection Kit (Roche) was applied to measure apoptotic nuclei. Nuclei were co-stained with TUNEL and DAPI. Cells were then visualized under a confocal microscope (Leica). For each treatment, 1000 cells in five random views (1×100 magnification) were counted to calculate the average TUNEL ratio (% vs. DAPI).

### ROS assay

Neuronal cells were seeded into six-well plates. Following treatment, cells were stained with fluorescent dye CellROX (Sigma, 7.5 μM for 1h). CellROX intensity was tested by a fluorescence spectrofluorometer (Molecular Devices, San Jose, CA, USA). Representative CellROX fluorescence images were presented.

### Glutathione (GSH) contents

Following treatment, a GSH/GSSG assay kit (Beyotime, Wuxi, China) was utilized to calculate the ratio of reduced glutathione to oxidized GSSG (GSH/GSSG×100%).

### DNA breaks

Neuronal cells were seeded into six-well plates. Following treatment, single strand DNA (ssDNA) contents, indicating DNA breaks, were measured through ssDNA ApoStrandTM ELISA kit (BIOMOL International, Plymouth Meeting, PA, USA). ELISA OD was examined at 450 nm.

### CRISPR-Cas9-induced p38α knockout (KO)

A CRISPR-Cas9-p38α-KO-GFP-puromycin construct was designed by Genechem (Shanghai, China) and transfected into cultured SH-SY5Y cells (in polybrene medium). GFP-positive SH-SY5Y cells were sorted by FACS and distributed to 96-well plates to achieve single cells. Stable cells were further selected by puromycin. In the stable cells, p38α KO was verified by Western blotting. The target DNA sequence of p38α sgRNA is *TGGACGTTTTTACACCTGCA* (PAM: *AGG*).

### Mitochondrial

immunoprecipitation (Mito-IP)**.** As described [25], following the applied treatment, a “Mitochondria Isolation Kit for Cultured Cells” (Pierce, Rockford, IL) was utilized to isolate mitochondria of neuronal cells (via high-speed centrifugation). The resulting mitochondrial fraction lysates (300 μg) were pre-cleared and incubated with anti-Cyclophilin D (CyPD) antibody [35]. Afterwards, protein IgG beads (30 μL per treatment, Sigma) were added to obtain CyPD-immunoprecipitated proteins, which were then tested by Western blotting. The mitochondrial CyPD-ANT1 (adenine nucleotide translocase 1)-p53 association was analyzed.

### Mitochondrial depolarization

JC-1 fluorescence dye aggregates into mitochondria to form green monomers in cells with mitochondrial depolarization [36]. Neuronal cells were seeded into 12-well plates. Following treatment, cells were stained with JC-1 (15 μg/mL, Sigma), and then washed and examined under a fluorescence spectrofluorometer (F-7000, Hitachi, Japan) at 488 nm (green). The representative JC-1 fluorescence images integrating both green (at 488 nm) and red (at 625 nm) fluorescence channels were presented as well.

### Annexin V-FACS

Neuronal cells were seeded into six-well plates at 1×10^5^ cells per well. With the applied treatment, cells were co-stained with Annexin V (15 μg/mL) and Propidium Iodide (PI, 15 μg/mL), and measured under a FACS machine (BD, Shanghai, China). Annexin V-positive cells (apoptotic cells) were gated and its ratio was recorded.

### Statistics

Data were expressed as means ± standard deviation (SD). Statistical analyses among different groups were tested by one-way analysis of variance (ANOVA) and Tukey’s post hoc multiple comparison tests (SPSS 23.0, SPSS, Chicago, IL, USA). The Student t test (Excel2007) was applied to compare statistical difference between two groups. P< 0.05 was considered as statistically significant.
